# Insights into mycobacteriome composition in *Mycobacterium bovis*-infected African buffalo (*Syncerus caffer*) tissue samples

**DOI:** 10.1038/s41598-024-68189-x

**Published:** 2024-07-30

**Authors:** Giovanni Ghielmetti, Tanya J. Kerr, Netanya Bernitz, Sinegugu K. Mhlophe, Elizma Streicher, Andre G. Loxton, Robin M. Warren, Michele A. Miller, Wynand J. Goosen

**Affiliations:** 1 South African Medical Research Council Centre for Tuberculosis Research, Division of Molecular Biology and Human Genetics, Faculty of Medicine and Health Sciences, Stellenbosch University, PO Box 241, Cape Town, 8000 South Africa; 2 Section of Veterinary Bacteriology, Institute for Food Safety and Hygiene, Vetsuisse Faculty, University of Zurich, Winterthurerstrasse 270, 8057 Zurich, Switzerland; 3 Cryptosporidiosis Lab, The Francis Crick Institute, London, UK

**Keywords:** African buffaloes, Culture-independent, Mycobacteriome, *Mycobacterium bovis*, Oxford nanopore technology, Targeted next-generation sequencing, Infectious-disease diagnostics, Pathogens

## Abstract

Animal tuberculosis significantly challenges global health, agriculture, and wildlife conservation efforts. Mycobacterial cultures are resource-intensive, time-consuming, and challenged by heterogeneous populations. In this study, we employed a culture-independent approach, using targeted long-read-based next-generation sequencing (tNGS), to investigate the mycobacterial composition in 60 DNA samples extracted from *Mycobacterium bovis* infected culture-confirmed African buffalo tissue. We detected mycobacterial DNA in 93.3% of the samples and the sensitivity for detecting *Mycobacterium tuberculosis* complex (MTBC) was 91.7%, demonstrating a high concordance of our culture-independent tNGS approach with mycobacterial culture results. In five samples, we identified heterogenous mycobacterial populations with various non-tuberculous mycobacteria, including members of the *Mycobacterium avium* complex (MAC), *M. smegmatis*, and *M. komaniense*. The latter *Mycobacterium* species was described in South Africa from bovine nasal swabs and environmental samples from the Hluhluwe-iMfolozi Park, which was the origin of the buffalo samples in the present study. This finding suggests that exposure to environmental mycobacteria may confound detection of MTBC in wildlife. In conclusion, our approach represents a promising alternative to conventional methods for detecting mycobacterial DNA. This high-throughput technique enables rapid differentiation of heterogeneous mycobacterial populations, which will contribute valuable insights into the epidemiology, pathogenesis, and microbial synergy during mycobacterial infections.

## Introduction

Animal tuberculosis (TB), caused by *Mycobacterium bovis* (*M. bovis*) and other members of the *Mycobacterium tuberculosis* complex (MTBC), is a threat to livestock and wildlife populations^1,2^. Among the different mycobacterial species, *M. bovis* is a significant pathogen capable of causing severe chronic infectious disease, particularly in animals such as African buffaloes (*Syncerus caffer*)^3^. African buffaloes are well-known wildlife maintenance hosts for *M. bovis*, contributing to the potential direct and indirect transmission of the pathogen to domestic livestock, other susceptible wildlife species like African lions (*Panthera leo*), rhinoceros (*Ceratotherium simum*, *Diceros bicornis*), African elephants (*Loxodonta africana*), and humans^4–7^.

In African buffaloes, *ante-mortem* detection of infection is based on the in vivo or in vitro measurement of *M. bovis* antigen-specific cell-mediated immunological (CMI) responses, using the tuberculin skin test (TST) or the interferon-γ (IFN-γ) release assay (IGRA), respectively^8–10^. Although conventional TST uses purified protein derivatives (PPDs) from *M. bovis* and *M. avium*, in vitro cytokine stimulation assays have employed PPDs or mycobacterial peptides, such as early secretory antigen target 6 kDa (ESAT*-6*) and culture filtrate protein 10 kDa (CFP-10), for identifying infected individuals. Unfortunately, cross-reactive host responses to non-tuberculous mycobacteria (NTMs), especially when using PPDs to stimulate antigen-specific cell-mediated immunity (CMI) responses, lead to diagnostic interference^11^. Except for well-documented pathogenic members, such as *M. leprae*, *M. marinum*, and *M. avium* subsp. *paratuberculosis*, NTM—previously regarded as benign environmental organisms prevalent in soil and water—are now recognized as potential pathogens with significant implications for human and animal health, particularly in immunocompromised individuals^12^. Infection with NTMs can cause diseases collectively referred to as mycobacteriosis, affecting not only humans but also various livestock and wildlife species^11,13,14^.

Currently, the gold standard for definitive confirmation and characterisation of TB relies on culture-based methods, where bacterial isolates obtained from infected animals undergo phenotypic and genotypic analyses^15^. However, the application of mycobacterial culture may not be feasible, especially in certain epidemiological contexts where the movement of animal samples is restricted. In South Africa, most of the Foot-and-Mouth Disease (FMD)-endemic areas coincide with well-known *M. bovis*-endemic wildlife parks. In these regions, the movement of cloven-hooved animals including samples is subjected to regulatory restrictions. As a result, there is a need for DNA-based culture-independent *M. bovis* diagnostic techniques as well as an unbiased approach to investigate complex host-mycobacterial interactions.

To date, a few commercially available real-time PCR assays have been reported to detect MTBC members and NTMs directly from animal specimens, including oronasal swabs, tissue homogenates, and respiratory secretions, including bronchoalveolar lavage samples. Assays include but are not limited to the Xpert^®^ MTB/RIF Ultra (Cepheid, Sunnyvale, California, USA)^16^, artus^®^ *M. tuberculosis* PCR Kit (Qiagen, Venlo, Limburg, Netherlands)^17,18^, COBAS^®^ TaqMan^®^ MTB Test kit (Roche Diagnostics, Indianapolis, Indiana, United States)^19^, BactoReal^®^ kit (Ingenetix, Vienna, Austria)^20^, and VetMAX™ *M. tuberculosis* Complex PCR Kit (Thermo Fisher Scientific, Waltham, Massachusetts, United States)^21,22^ for MTBC DNA detection, and the Hain Geno‐Type CM*direct* VER 1.0-line probe assay^5^ for MTBC/NTM DNA detection and identification of commonly found NTM species in human clinical samples. However, these assays cannot differentiate between the different members of the MTBC, in addition, the Hain Geno‐Type CM*direct* VER 1.0-line probe assay has been reported to be less successful when applied to samples that had not undergone prior culture^5^. In contrast, PCR amplification of housekeeping genes such as the beta subunit of RNA polymerase (*rpoB)*, partial heat-shock protein (*hsp65)*, and the *Ku* genes, in conjunction with amplicon sequencing, have successfully detected *Mycobacterium* genus DNA and provided species identification in *ante-mortem* samples from wildlife species, circumventing the need for culture-based methods^5^. However, these targets are unable to differentiate specific MTBC members and require an additional region-of-difference PCR (RD-PCR) for speciation^23^. Unfortunately, RD-PCRs were initially tailored for the identification of MTBC in mycobacterial cultures, posing significant challenges in adapting them for use with DNA extracted directly from samples^23^. Sequencing of specific genetic targets has shown promise as an alternative tool for discerning phylogenetically related slow-growing mycobacteria by interrogating DNA sequence polymorphisms^24^. Genes encoding DNA gyrase subunits *gyrA* and *gyrB* have demonstrated satisfactory discriminatory power, facilitating precise taxonomic resolution and classification of distinct MTBC members^25,26^.

The 16S rRNA gene is widely used for bacterial identification, aiding in the discovery of new species and serving as an alternative to traditional identification methods. Direct sequencing of amplified 16S rRNA gene DNA in *Mycobacterium* species is well-documented^27,28^. Over the past decade, however, several authors suggested that the inter- and intraspecies discriminatory power of 16S rRNA gene sequences was insufficient for some bacterial genera, including *Mycobacterium* species^29,30^. Despite being a sub-optimal gene target for *Mycobacterium* genus speciation, studies have demonstrated the advantages of using full-length 16S rRNA amplicon sequencing for taxonomic classification, as opposed to short-read amplicon sequencing^31–33^. Nonetheless, previous methods for high-throughput full-length 16S rRNA gene amplicon sequencing using Oxford Nanopore Technologies (ONT) technology often relied solely on reference database alignment^34,35^ rather than employing de novo generation of sequence features, such as amplicon sequence variants (ASV) or operational taxonomic units (OTU).

Currently, the generation of whole-genome sequences for epidemiological investigations of animal TB relies on mycobacterial culture; however, analyses can be impeded by the presence of other microorganisms that outcompete MTBC growth in culture^36–38^. Over the past decade, an increasing number of publications have focused on direct whole genome sequencing (WGS) using hybridization-based target enrichment techniques to bypass mycobacterial culture and generate high-quality MTBC genomes directly from infected tissue samples^39,40^. However, their current considerable commercial cost limits their application in the veterinary field in low-income settings, especially when analyzing large numbers of samples. Furthermore, the presence of genetically closely related microorganism communities in samples poses a challenge for de novo whole genome assembly^41^. Therefore, identifying these communities before sequencing is crucial.

Co-infections can also modulate the immune response, particularly in infections with closely related microorganisms that share antigenic properties^42^. This study describes the use of three housekeeping genes (*hsp65*, *rpoB,* and 16S rRNA) in a culture-independent in-house multiplex PCR method to detect and identify *Mycobacteria* spp., including MTBC, followed by single-nucleotide polymorphism (SNP) for speciation of the MTBC (*gyrA* and *gyrB*), directly from culture-confirmed *M. bovis* infected tissue.

Using a subset of *M. bovis* infected tissue samples from African buffaloes, a novel culture-independent targeted next-generation sequencing (tNGS) approach has been recently developed^43^. In this study, we aimed to characterize the mycobacteriome, which refers to the community of mycobacteria present in a sample, of a larger group of samples obtained from the same animal species and geographical region. For this purpose, we applied a novel reference-free sorting tool^44^ on ONT-sequenced amplicons and based on their similarity in sequence and length, we were able to build consensus sequences revealing the mycobacteriome present in each sample^45^. This approach contributes to the refinement of diagnostic strategies, enhancing our ability to comprehensively study the mycobacterial landscape in wildlife and ensuring the effective surveillance of *M. bovis* in regions where it poses a significant threat to both animal health and conservation efforts.

## Results

A total of 60 tissue samples from 57 individual African buffaloes^43^ from the Hluhluwe-iMfolozi Park (HiP), South Africa (SA), were selected in the present study and included pooled head lymph nodes (n = 5), pooled thoracic lymph nodes (n = 7), lung tissue (n = 7), tonsil (n = 5), tracheobronchial lymp nodes (n = 12), mediastinal lymph nodes (n = 8), parotid (n = 4), retropharyngeal lymph nodes (n = 6), prescapular lymph nodes (n = 3), two mandibular lymph nodes, and one subiliac lymph node. All buffalo samples had previously been confirmed as infected using the aforementioned culture methods, and the presence of the *M. bovis* region of difference 4 (RD4) signature was confirmed from culture-positive crude DNA extracts^23^. Spacer oligonucleotide typing hybridization assay (spoligotyping) revealed that all samples fit one of two profiles, SB0130 (n = 39) and SB1474 (n = 21) (Suppl. Table 1).

### ONT targeted amplicon sequencing

**Figure 1:**
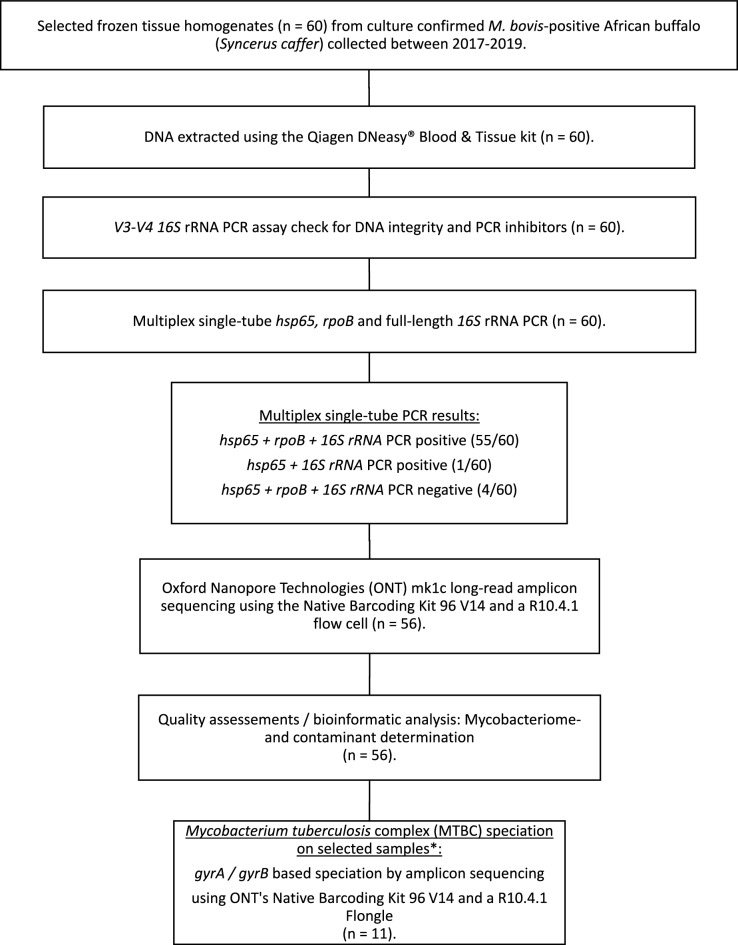
Study overview illustrating the methods applied to DNA extracted from culture-confirmed *M. bovis*-positive tissue samples (n = 60) from 57 different African buffalo, selected and included in the study following mycobacterial species identification. After amplifying three target genes (*hsp65*, *rpoB*, 16S rRNA), long-read amplicon sequencing was conducted on 56 samples using Oxford Nanopore Technologies (ONT), the Native Barcoding Kit 96 V14, and a single R10.4.1 flow cell. *Mycobacterium tuberculosis* complex (MTBC) speciation was achieved by amplifying and sequencing the *gyrA* and *gyrB* gene targets on 11 randomly selected samples using a single Flongle R10.4.1 flow cell (ONT).

**Figure 2:**
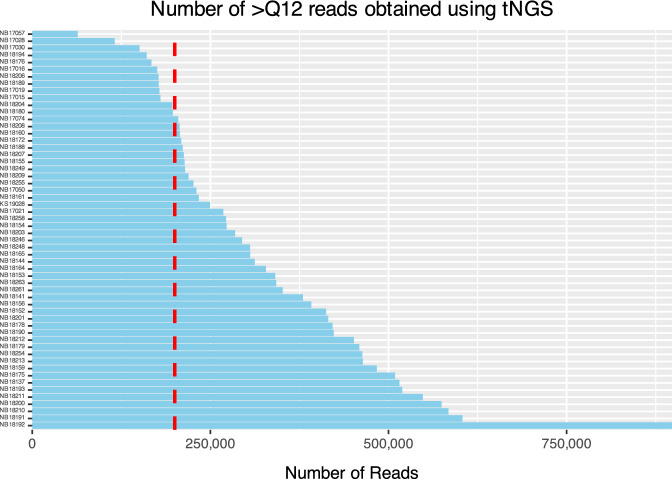
Number of reads with quality score > Q12 obtained using Oxford Nanopore Technologies (ONT) targeted next-generation sequencing (tNGS) across 56 African buffalo tissue samples. Among 78.6% of the samples (n = 44), over 200,000 reads were generated, with a maximum of 897,809 reads. Conversely, the remaining 21.4% (n = 12) of samples yielded reads ranging between 63,705 and 197,343, all of which were included in the analysis. The vertical red dotted line highlights the threshold for the number of reads used for downstream analysis.

All samples showing amplification of > 1 target gene (n = 56; 93.3%) were included for amplicon sequencing, whereas four samples (2 tonsils, one lung, and one parotid) for which none of the targets could be visualized after electrophoresis were excluded from downstream analysis. The *hsp65, rpoB*, and 16S rRNA gene regions were successfully amplified from 55 samples (Fig. 1). One pooled head lymph node sample did not amplify the *rpoB* target. The number of reads per specimen with Q12 or greater ranged from 63,705 to 897,809 (Mean (*M*) = 320,985 reads, Standard Deviation (SD) = 154,129), and the total number of bases sequenced ranged from 38,921,972 to 554,606,081 (*M* = 199,128,119 bases, SD = 95,521,803). The N50 read length (median read length of the longest contigs) varied from 558 to 781 bases. The mean read length for the samples ranged from 576 to 658 bases. The mean read quality, as indicated by the Phred score, ranged between 14.2 and 14.5. A total of 200,000 reads with > Q12 were randomly selected from each sample for downstream analysis (Fig. 2).

**Figure 3:**
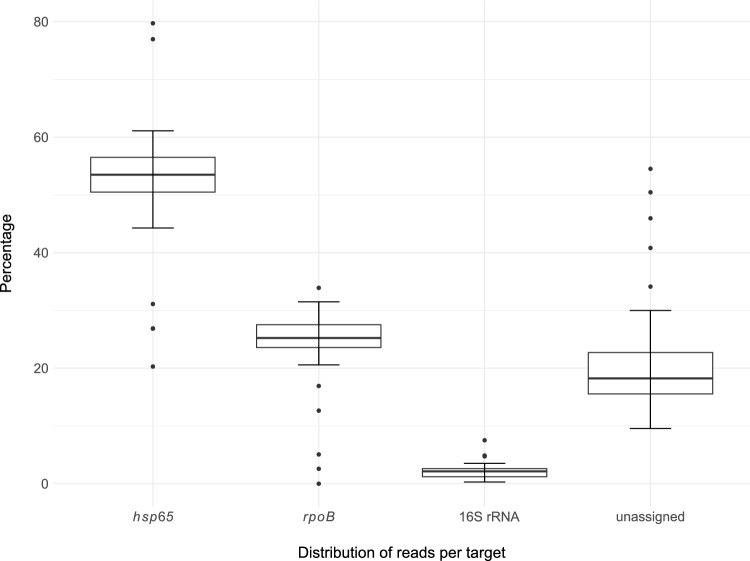
Distribution plot of read counts for the 56 African buffalo tissue samples and each genetic target (*hsp65*, *rpoB*, and 16S rRNA). Reference-free sorting and assembly of consensus sequences were performed on a maximum of 200,000 randomly selected reads (> Q12) for each sample. For tissue samples with fewer reads, the entire available dataset was included. Reads were generated using Oxford Nanopore Technologies (ONT) on selected targeted genes shown on the x-axis. Singletons that could not be included in any group because of their length or sequence similarity are reported as unassigned and were excluded from the analysis.

Since amplification of the three specific genetic targets occurred in a single tube, amplified PCR products were not normalized, and therefore shorter sequences were predominant. The proportion of reads for each target varied, with *hsp65* ranging from 29 to 80% (*M* = 52.6%; mean number of reads 101,132), *rpoB* 2–34% (*M* = 24.4%; mean number of reads 47,325), and 16S rRNA 0.3–8% (*M* = 2.2%; mean number of reads 4,007) (Fig. 3). Singletons that could not be assigned to any group because of their length or sequence similarity were reported as unassigned 9–54% (*M* = 20.8%; mean number of reads 39,330) and excluded from the downstream analysis.

Using a subset of eleven tissue DNA samples, the g*yrA* and g*yrB* genes were amplified and sequenced on a single Flongle flow cell (ONT). The number of reads with Q12 or greater ranged from 118 to 1918 (*M* = 1157 reads, SD = 489), and the total number of bases sequenced ranged from 20,411 to 328,224 (*M* = 200,948 bases, SD = 85,764). The mean read quality, as indicated by the Phred score, was consistently above 13 (11/11). All reads with > Q12 quality scores were selected from each sample for downstream analysis. Specifically, the *gyrA* amplicon sequences confirmed the presence of MTBC DNA in approximately three-quarters of the amplified samples (8 out of 11), while *gyrB1* and *gyrB2* confirmed the RD and spoligotyping speciation results obtained from culture-derived DNA in all samples.

### Mycobacterial DNA detection and mycobacteriome composition

**Figure 4:**
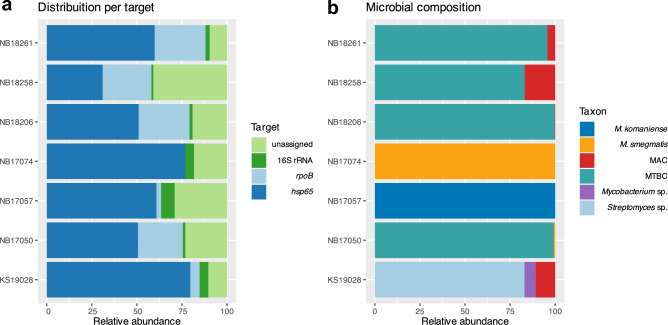
(**a**) Visualization depicting the percentage of reads across different target genes (16S rRNA, *rpoB, hsp65*) for DNA from each African buffalo tissue sample (n = 7) where the presence of non-tuberculous mycobacteria or heterogeneous mycobacterial communities was detected. Reference-free sorting and assembly of consensus sequences were performed on randomly selected reads. The relative abundance of the target genes amplified using a multiplex PCR and sequenced using Oxford Nanopore Technologies is shown. (**b**) The relative abundance of reads appertaining to different mycobacteria and closely related organisms including *Streptomyces* sp. is illustrated. Identification of mycobacterial communities based on the amplification of the *hsp65* gene. *Mycobacterium tuberculosis* complex (MTBC); *Mycobacterium avium* complex (MAC); NB18261 mediastinal lymph node; NB18258 lung; NB18206 subiliac lymph node; NB17074 and KS19028 pooled head lymph nodes; NB17057 parotid; NB17050 retropharyngeal lymph node.

Evidence of *Mycobacterium* spp. DNA presence was detected in 93.3% (56/60) of the DNA samples extracted directly from tissue homogenates. Among the 56 samples subjected to amplicon generation and subsequent deep sequencing for the three specific genetic targets, the *rpoB* PCR exhibited the highest sensitivity 91.7% for MTBC detection (55/60), followed by *hsp65* 88.3% (53/60), and 16S rRNA 86.7% (52/60). Overall, the mycobacterial DNA detection and composition of the mycobacteriome varied depending on the three independent targets. The shorter target PCR (*hsp65*) was predominant (Fig. 4a). It demonstrated the highest efficacy in detecting NTM and heterogeneous mycobacterial populations compared to alternative targets (Table 1), as well as closely related microorganisms such as *Streptomyces* sp. (Fig. 4b). Overall, the presence of NTM and heterogeneous mycobacterial communities was identified in seven African buffaloes tissue samples, including one parotid (NB17057), two pooled head lymph nodes (NB17074 and KS19028), one retropharyngeal lymph node (NB17050), one subiliac lymph node (NB18206), one mediastinal lymph node (NB18261), and one lung (NB18258).

**Table 1:** Synopsis of the reference-free sorting and assembly outcomes of consensus sequences for the three *Mycobacterium* spp. target genes (*hsp65, rpoB*, 16S rRNA) included in the culture-independent next-generation sequencing approach.

	*hsp65*	*rpoB*	16S rRNA
*Mycobacterium tuberculosis* complex (MTBC)	49	54	52
*Non-tuberculous mycobacteria* (NTM)	3	0	2
MTBC + NTM	4	1	0
Total	56	55^a^	54^b^

The mycobacterial DNA detection and composition of the mycobacteriome varied depending on the three independent targets. Notably, the shorter target PCR (*hsp65*) demonstrated the ability to identify the highest proportion of mycobacteria, with amplification and *Mycobacterium* spp. sequences detected in 93.3% of the samples tested (56/60). The *rpoB* PCR displayed the highest sensitivity for MTBC detection, identifying MTBC in 91.7% of all samples included (55/60).

^a^One sample could not be amplified using the *rpoB* target.

^b^For two samples (NB18154 and KS19028), the 16S rRNA consensus sequences were classified as organisms not belonging to the *Mycobacterium* genus. Further analysis using NCBI Basic Local Alignment Search Tool (BLAST) identified the presence of *Niallia* sp. in NB18154 and *Streptomyces* sp. in KS19028.

Based on previous molecular analyses, which involved RD-PCRs and spoligotyping used to confirm the presence of *M. bovis* within the selected samples, our goal was to assess the ability of the novel culture-independent methodology to detect and speciate MTBC members. The three additional targets employed for confirming the presence of *M. bovis* DNA (*gyrA*, *gyrB1*, and *gyrB2*) were successfully amplified in all 11 selected samples. For two samples (NB17057 and KS19028), however, the total number of reads (656 and 118) and the number of consensus sequences identified as MTBC in one or more genetic targets (92 and 43) were lower compared to the corresponding mean values generated for the other samples (total number of reads 10,149).

**Figure 5:**
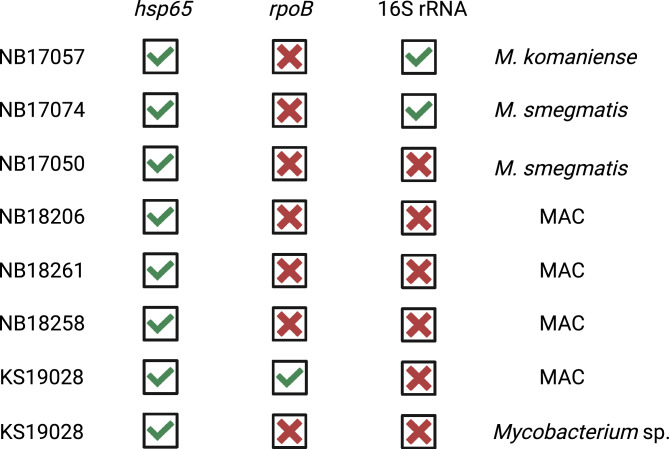
Agreement between different genetic targets in detecting and identifying non-tuberculous mycobacteria (NTM) in culture-confirmed *Mycobacterium bovis* infected African buffalo tissue sample (n = 7). The shorter target PCR (*hsp65*) demonstrated the highest efficacy in detecting NTM and heterogeneous mycobacterial populations compared to alternative targets. The latter target identified eight NTM in seven samples, *rpoB* one member of the *Mycobacterium avium* complex (MAC), and 16S rRNA detected two NTM in distinct samples. In sample KS19028 (bottom two rows), obtained from pooled head lymph nodes, a member of the MAC and an unclassified *Mycobacterium* sp. were detected in addition to *M. bovis*. A complete consensus of results was observed across different target PCRs when NTM were detected by two separate genes.

In addition to the detection of MTBC DNA, tNGS of the *hsp65* gene successfully identified different mycobacterial species in various tissue specimens, including two pooled head lymph nodes, one lung, one mediastinal lymph node, one parotid, one retropharyngeal lymph node, and one subiliac lymph node. *Mycobacterium avium complex* (MAC) was detected in 6.7% samples (4/60), *M. smegmatis* in 3.3% (2/60), *M. komaniense* and an unclassified *Mycobacterium* sp. in one sample each (1.7%). Additionally, DNA of mycobacteria’s closely related organisms such as *Streptomyces sp.* were amplified and further identified using the *hsp65* target gene (Fig. 4b). In the DNA sample NB17074, originating from lung tissue, *M. smegmatis* was identified using *hsp65* and 16S rRNA targets, but no amplification was observed using *rpoB* PCR (Fig. 5). Conversely, in one retropharyngeal lymph node (sample NB17057), *M. komaniense* was detected using *hsp65* and 16S rRNA PCRs, while MTBC DNA was detected using *rpoB*. For the three samples where NTM were detected by more than one target PCR—*M. komaniense* and *M. smegmatis* (*hsp65* and 16S rRNA) and a member of the MAC (*hsp65* and *rpoB*)—a 100% agreement between the different target results was observed (Fig. 5).

At least two distinct strains of MAC were detected, exhibiting 15 genomic modifications, including single nucleotide variations and indels distributed over the 441 bp of the *hsp65* amplicon. These strains displayed the highest *hsp65* sequence homology to *M. avium* and *M. colombiense* and possibly represent different strains of yet-to-be-characterized species. The MAC sequences were exclusively detected in tissue samples exhibiting heterogeneous bacterial populations (4/4), including MTBC in three samples, and a combination of *Streptomyces* sp. and *Mycobacterial* sp. in one sample (Table 2). The samples originated from various locations including lung tissue, mediastinal lymph nodes, head lymph nodes, and subiliac lymph nodes. Finally, the relative abundance of reads classified as MAC varied between 0.3 and 17% for the *hsp65* target in the above-mentioned samples (Fig. 4b).

**Table 2:** Percentage of *hsp65* PCR target reads (%) indicating the presence of non-tuberculous mycobacteria or heterogeneous mycobacterial communities in each African buffalo tissue sample where the DNA of these organisms was detected.

	NB17057 Parotid	NB17074 Head Lnn	NB17050 Retrop Ln	NB18206 Subiliac Ln	NB18261 Mediast Ln	NB18258 Lung	KS19028 Head Lnn
*M. komaniense*	100	0	0	0	0	0	0
*M. smegmatis*	0	100	0.86	0	0	0	0
MTBC	0	0	99.14	99.68	95.7	83.01	0
MAC	0	0	0	0.32	4.3	16.99	10.9
*Mycobacterium *sp.	0	0	0	0	0	0	6.13
*Streptomyces *sp.	0	0	0	0	0	0	82.97

Lymph nodes (Lnn); retropharyngeal lymph node (Retrop Ln); mediastinal lymph node (Mediast Ln).

## Discussion

While *M. bovis* culture is regarded as the gold standard technique for detecting animal TB, it necessitates a substantial bacterial load and is time-intensive, often taking eight weeks or more to confirm a diagnosis definitively^15,46^. To overcome these challenges, various direct molecular techniques, such as real-time PCR, have been developed for rapid and specific identification of MTBC DNA^40^. The advantages of this approach include high diagnostic sensitivity (Se), specificity (Sp), and shorter turnaround time for a definitive diagnosis. For instance, recent studies reported Se varying between 87.70 and 100%^21,47–49^ and Sp of 93.66–100%^49–52^ for targeted PCR detection of *M. bovis* in tissue samples from infected cattle herds compared to mycobacterial culture. However, while direct molecular techniques enable rapid and accurate diagnosis, they require careful design and validation to avoid false positive results due to imperfect specificity. Cross-reactivity of IS*6110*-based real-time PCRs, which is one of the most commonly used genetic targets for MTBC detection, has been reported for non-MTBC mycobacterial isolates, such as *M. marinum, M. wolinskyi,* and specific lineages of “*M. avium* subsp*. hominissuis”*. At the same time, MTBC strains lacking commonly used real-time PCR target genes such as IS*6110* have been described from human clinical samples, leading to false negative outcomes^53,54^. Therefore, despite their advantages, the design and validation of real-time PCR-based detection of MTBC in animals strongly depend on factors such as the target population, genetic target, and epidemiological situation. These considerations are crucial when employing direct molecular techniques for *M. bovis* DNA detection and animal TB surveillance, given the vast and complex nature of MTBC molecular detection and diagnosis.

Our culture-independent detection method applied to 60 samples, independently confirmed to harbour *M. bovis* through culture and molecular characterisation (RD-PCRs and spoligotyping), showed a Se of 91.7%, which was comparable to previous observations using different molecular means^21,47–49^. The majority of samples exhibited visible lesions (VL), with one-third of the animals (n = 30) being heavily infected and showing prominent lesions with scores of 2–3. However, over 20% (n = 13) exhibited no visible lesions (NVL), which are known to be more susceptible to false-negative results in culture^46^. The lower culture-positive rates of NVL reactors may be attributed to the low bacterial load in tissues and the detrimental effects of decontamination, which can potentially reduce the recovery of bacilli^46^. Notably, in the present study, 10/13 of the animals (16.7% of the entire dataset), had NVL and performed well using the mycobacteriome assay, leading to the amplification of mycobacterial-specific amplicons. It is promising to observe that, among these 10 samples, *M. bovis* DNA was detected in at least one target in nine samples. In contrast, in the remaining sample, only *M. smegmatis* was detected using *hsp65* and 16S rRNA. Finally, of the four samples (2 tonsils, one lung, and one parotid) for which none of the targets could be visualized after electrophoresis, three were NVL samples and one presented a grade 1 lesion.

The advantage of the approach reported in this study is the ability to distinguish between various mycobacterial species and, with careful consideration due to differences in primer affinities estimate the relative abundance of each population present in one sample, facilitating characterisation of the mycobacteriome. This approach targets a wide range of mycobacterial species, which may lead to interference in culture or in vivo diagnostic test results. Furthermore, the single-tube amplification of multiple genetic targets allows the user to obtain a more comprehensive understanding of the sample composition. However, caution is advised when interpreting the abundances of amplicons obtained for the three target genes, as the affinity of the primer pairs used may vary significantly. Finally, the inclusion of discriminatory genes, such as *gyrA* and *gyrB*, provides additional information, especially for complex clinical settings, where mixed infections may occur in various hosts and the environment^55–61^. Mycobacterial species differentiation can have significant implications for antimicrobial treatment of zoonotic TB, since most *M. bovis* strains are naturally resistant to pyrazinamide, an essential first-line antibiotic used to treat TB patients^62^.

This study explores the capabilities of long-read Nanopore-based sequencing to provide high-throughput and high-accuracy profiles of housekeeping genes for the genus *Mycobacterium*, alongside de novo generated consensus sequences utilizing full-length 16S rRNA gene amplicon sequencing in the context of animal TB. The presence of NTM in animals and people infected with MTBC carries significant medical consequences. Firstly, their presence may hinder the detection of MTBC through culture methods, potentially resulting in false negative outcomes. Secondly, NTM could impact immune responses to MTBC, influence the progression of infection, and modulate host response to existing live attenuated vaccines such as the Bacillus Calmette–Guérin (BCG) strain^63–65^.

The most common NTM found in this study through targeted deep sequencing were members of the MAC, followed by *M. smegmatis*, *M. komaniense*, and an uncharacterized *Mycobacterium* sp. This finding aligns with previous observations in wild and domestic ungulates such as cattle^14,66^, pigs^67^, wild boar^68^, and African buffalo^13^, suggesting a high presence of *M. avium* and *M. colombiense* in tissue samples due to transient colonisation or co-infection with MTBC. In this study, *M. komaniense*, a rapidly growing NTM closely related to *M. moriokaense*, was identified in a single parotid showing a lesion score of 2. Notably, this newly described mycobacterial species was initially documented in bovine nasal swabs, soil, and water samples collected from 2010 to 2012 in South Africa^69^. One of the four isolates previously reported by Gcebe et al. was obtained from a soil sample taken in the same geographic region (HiP, KwaZulu Natal Province) where the buffaloes in the current study were sampled^69^. These findings suggest potential geographic adaptation of certain mycobacterial species and temporal persistence. However, the clinical significance of *M. komaniense* is unknown. The concurrent detection of *M. bovis* and *M. komaniense* in a retropharyngeal lymph node exhibiting a small focal lesion with a diameter < 10 mm (lesion score 1) does not necessarily preclude the possibility of an incidental finding. With the rising incidence of NTM infections, particularly those caused by MAC, among both immunocompromised and immunocompetent human patients globally, there is a need for further investigation of the clinical implications posed by these emerging pathogens. A One Health approach, that includes mycobacterial strains from human, animal, and environmental origins, is required to investigate common etiological determinants of infection, comorbidities, and virulence factors influencing clinical outcomes.

In this study, a targeted PCR deep-sequencing culture-independent approach, using DNA from tissues confirmed to contain *M. bovis*, was used to explore the composition of the mycobacteriome. The shorter target PCR (*hsp65*) demonstrated superior capability in detecting NTM and heterogeneous populations of mycobacteria compared to alternative targets. However, since none of the three specific genetic targets amplified simultaneously in the multiplex PCR could distinguish between *M. bovis* and other members of the MTBC using amplicon sequencing, the incorporation of additional ecotype-specific markers (g*yrA* and g*yrB* genes) was imperative for the diagnostic pipeline^70^. Implementing the outlined method in future studies, particularly those involving samples containing multiple mycobacterial species and from microbially complex sites, such as the upper respiratory tract, will enhance understanding of the diverse interactions and within-host coexistence of mycobacterial species.

One of the main limitations of the study is the lack of confirmation for the presence of NTM in MGIT cultures, complicated by the concomitant growth of *M. bovis*. Further studies investigating heterogeneous mycobacterial populations using selective culture media will enable the determination of NTM viability in *M. bovis*-affected animals and assess the ability of different target PCRs in detecting NTM compared to culture. Another limitation of the mycobacteriome approach is that mycobacteria-specific primers may encounter more amplification difficulties with the targeted genes compared to the more conserved targets used in metagenomic investigations^71^. To address this issue, we used multiple targets. The observed differences in amplification between these targets likely reflect their varying affinities for the species detected.

Similar to prior research reports^72–74^, our findings indicate that Nanopore-based targeted amplicon sequencing can generate comparable results to existing high-throughput assays for the detection and characterisation of MTBC organisms. However, it is important to note that Nanopore costs may fluctuate significantly based on batch sizes and the utilization of flow cells. Moreover, because of the frequent updates in chemistry and base calling algorithms, constant validation of new kits and pipelines might pose a hurdle for clinical applications. One of the main drawbacks is the short longevity of flow cells, which may impede progress in longer-term projects. Finally, transitioning entirely from mycobacterial cultures, which usually enhance DNA yield and indicate viability, poses a challenge in demonstrating the transmission and actual infection potential of the mycobacteria detected via a culture-independent approach. Both culturing and PCR may introduce bias, leading to findings that might not faithfully reflect the original mycobacteriome^75,76^. Therefore, innovative research efforts should focus on refining RNA-based detection methods to differentiate viable bacilli and remnant DNA draining through the lymph system in clinical samples^77^.

## Conclusion

By applying a culture-independent targeted next-generation sequencing approach to DNA extracted from previously culture-confirmed *M. bovis* infected African buffalo tissues, we were able to detect mycobacterial DNA in 93.3% of the samples. Of these, 98.2% were positive for MTBC DNA. Mycobacterial-specific amplicons were sequenced and identified positive tissue samples using a one-tube multiplex amplicon sequencing approach. This technique provided rapid and comparable results to previously published molecular methods. This study presents a promising alternative to culture-based detection methods for the detection of animal TB and for characterisation of mycobacterial communities in wildlife tissue samples.

## Materials and methods

### Study population

Between 2017 and 2019, a routine veterinary annual test-and-slaughter program was conducted in Hluhluwe-iMfolozi Park (HiP), South Africa (SA), on different herds of African buffaloes^3,10^. All animals were captured, immobilized, and whole blood collected, as previously described^9^. Positive animals were euthanized by gunshot based on the detection of cell-mediated immunity (CMI) responses toward *M. bovis-*specific antigens (n = 67). All buffaloes were handled by the Enzemvelo Wildlife Services and KZN state veterinarians. Various tissue samples (lymph nodes from the head, thorax, and lung) were collected during necropsies, and lesion scores were assigned as previously described (Suppl. Table 1)^8^. For each sample, tissue homogenate aliquots were split for mycobacterial culture and culture-independent analyses, with processing occurring in a BSL-3 laboratory up to the step of DNA extraction. Briefly, approximately 10 g of tissue was homogenized in 50-mL skirted tubes (Becton Dickinson, Franklin Lakes, New Jersey, USA) containing eight 4.8-mm metal beads and 4 mL of sterile phosphate-buffered saline (PBS) using a blender (Bullet Blender 50; Next Advance, Averill Park, NY, USA) for 15 min at maximum speed, as previously described^78^. After decontamination, performed with BD MycoPrep™ (N-acetyl L-cysteine sodium hydroxide) and PBS following the manufacturer’s instructions, all samples were centrifuged for 15 min at 1500×*g* and the supernatant was decanted. Each pellet was resuspended in 1 ml PBS and 500 μl of this suspension was transferred to a Mycobacteria Growth Indicator Tube (MGIT™) supplemented with PANTA and incubated in a BACTEC™ MGIT™ 960 Mycobacterial Detection System (both Becton Dickinson). An additional aliquot of tissue homogenate was subsequently preserved (− 80 °C) for DNA extractions, repeat culturing, and/or future sequencing. All Mycobacteria Growth Indicator Tubes (MGIT™) were incubated for 56 days and culture-positive crude extracts were subjected to speciation using a PCR targeting genetic regions of difference to confirm *M. bovis* infections, as previously described^23^. Further genetic speciation using spoligotyping was performed^79^.

### Sample selection and processing

A total of 60 frozen native tissue homogenates from 57 individual African buffaloes, including seven animals previously involved in the development of the mycobacteriome assay^43^, were retrospectively selected from the cohort mentioned above and included for downstream analysis (Fig. 1) based on *M. bovis* culture and PCR confirmation. A total of 78.3% (47/60) of the samples exhibited macroscopic lesions, with one-third (30/60) classified with lesion scores of 2–3. Previously frozen tissue homogenates (1 mL aliquot) were subjected to genomic DNA extraction using the DNeasy Blood and Tissue kit (Qiagen) with modifications, as previously described^43^. Briefly, tissue homogenates were heat-inactivated (98 °C for 45 min), then centrifuged (1500×*g* for 10 min), after which 300 μL of Buffer ATL (Qiagen) was added to the cell pellet. Subsequently, 25 μL of Proteinase K (Qiagen) was added and allowed to digest overnight at 56 °C with agitation at 600 rpm. The sample was centrifuged at 5500×*g* for 5 min, after which 500 μL of supernatant was collected and transferred to a 1.5 mL tube. An additional 400 μL of buffer AL and 400 μL of ethanol was mixed with the supernatant and then transferred to a Mini Spin Column (Qiagen). Finally, DNA purification was conducted using wash buffers AW1 and AW2 (Qiagen), followed by elution in 60 μL of buffer AE (Qiagen) pre-warmed to 54 °C. Concentrations of DNA were quantified using the Qubit 1 × dsDNA High Sensitivity Assay kit (Thermo Fisher Scientific), following the manufacturer's instructions. Subsequently, DNA integrity and presence of PCR inhibitors were assessed by amplification of the variable regions V3-V4 within the 16S gene, using previously published primers (prbac1/prbac2) and PCR conditions^80^. Finally, the PCR products were visualized by 1% agarose gel electrophoresis.

### Targeted amplicon sequencing using Oxford Nanopore Technologies (ONT)

A multiplex PCR-based amplification targeting three housekeeping genes, namely *hsp65* (441 bp), *rpoB* (680 bp) and the full-length 16S rRNA gene (~ 1500 bp), was performed culture-independently using DNA extracted from buffalo tissue homogenates, as previously described^43^. Presence of the amplified products was confirmed by 1% agarose gel electrophoresis. All samples showing amplification of > 1 target were included for amplicon sequencing. Using the Native Barcoding Kit 96 V14 kit (ONT), amplicons were end-repaired, individually barcoded, pooled into a single library, native adapter-ligated, loaded onto a single R10.4.1 flow cell (> 1250 pores), and sequenced on a MinION mk1C device (ONT). Building upon prior molecular analyses, which encompassed RD-PCRs and spoligotyping, confirming the presence of *M. bovis* within the selected tissues, we aimed to verify the efficacy of the novel culture-independent methodology on a subset of samples. Therefore, eleven DNA samples were randomly selected to confirm the presence of *M. bovis* using *gyrA* and *gyrB* targeted amplicon sequencing. Three independent 25 μL reactions containing 14 μL Q5 High-Fidelity 2X Master Mix (New England Biolabs Inc., Ipswich, Massachusetts, United States), 0.5 μL of each 50 μM primer stock solution^25^, 6 μL sterile, nuclease free water, and 2 μL undiluted extracted DNA. The PCR cycling conditions were as follows: 1 cycle initial denaturation at 98 °C for 15 min, followed by 40 cycles of denaturation (98 °C for 30 s), annealing (62.5 °C for 30 s) and elongation (72 °C for 2 min). Final elongation took place at 72 °C for 5 min. The presence of the amplified products was confirmed by 1% agarose gel electrophoresis. Amplicons were end-repaired, individually barcoded, and native adapter-ligated using the Native Barcoding Kit 96 V14 kit (ONT). Finally, the pooled library was loaded onto a single Flongle R10.4.1 flow cell (> 60 pores) and sequenced using the MinION mk1C device (both ONT).

### Data analyses

For the targeted amplicon sequencing datasets, base-calling, demultiplexing, and trimming of the barcodes were performed in real-time using Guppy [v6.4.6] (260 bps—High-Accuracy)^81^. Data acquisition and base-calling were stopped after 72 h for the three specific genetic targets run using an R10.4.1 flow cell and after 22 h for the *gyrA* and *gyrB* run using a Flongle flow cell (Supplementary Materials 1 and 2). Quality control, filtering, and summary reports for Nanopore reads were generated using nanoq v0.10.0^82^ and reads with a Q score of < 12 were discarded. Thereafter, reference-free reads sorting, based on similarity and length, was performed using the amplicon sorter tool [v2023-06-19]^44^. Since over 80% of the samples yielded more than 200,000 reads and computational analysis remained feasible, we opted to increase the number of reads compared to our previous study^43^. A total of 200,000 randomly chosen reads with minimum and maximum lengths of 300 bp and 2000 bp, respectively, were selected for each barcode generated, using the three target amplicon approach (*hsp65*, *rpoB*, and 16S rRNA). Finally, ABRicate (https://github.com/tseemann/abricate) and custom databases, generated as previously described^43^, were used for the screening of consensus sequences and summarizing the report files. For the analysis, the following interpretation criteria were established:

- Coverage ≥ 90% and identity ≤ 90%, the sequence was reported as unclassified.
- Coverage ≥ 90% and identity fell within the range of 90–98%, the sequence was classified as *Mycobacterium* sp.
- Coverage ≥ 90% and identity ≥ 98%, the sequence was reported according to the results table.

Since the database only contained mycobacterial sequences and to characterise bacterial contaminants, all consensus sequences with a corresponding length to 16S rRNA and showing an identity lower than 98% were manually searched using the NCBI Basic Local Alignment Search Tool (BLAST).

Reads generated from the *gyrB* and *gyrA* amplicons were selected based on lengths with a minimum of 50 bp to a maximum of 200 bp. Consensus sequences were then generated for each species amplified and target gene. Relative abundances were determined by analyzing the representative pool of reads. Sequence comparison with a custom database, generated with *gyrB* and *gyrA* sequences for all members of the MTBC, was performed as above.

For all bioinformatics tools, default settings were used unless stated otherwise. The proportion of consensus sequences generated per target gene and relative abundance of *Mycobacterium* sp. identified across different samples was visualized using the R package ggplot2.

### Ethics

South African Veterinary Council (SAVC)-registered wildlife veterinarians were responsible for all procedures, including immobilization of animals, blood collection, euthanasia, and tissue sampling. No animal was specifically immobilized, sampled, or sacrificed for this study. Ethical approval for this study was granted by Stellenbosch University Animal Care and Use Research Ethics Committee (SU-ACUD16-00072; SU-ACU-2019-9081) and the Stellenbosch University Biological and Environmental Safety Research Ethics Committee (SU-BEE-2021-22561). Section 20 approval was granted by the South African Department of Agriculture, Land Reform and Rural Development (DALRRD 12/11/1/7/6 and 12/11/1/7/2). The study was conducted following the local legislation and institutional requirements and the authors complied with the ARRIVE guidelines.

### Supplementary Information

Supplementary Information 1.

Supplementary Information 2.

Supplementary Table 1.

## Data Availability

The datasets presented in this study were submitted to the European Nucleotide Archive (ENA) under project reference number PRJEB75236, https://www.ebi.ac.uk/ena/browser/view/PRJEB75236.
