# Supplementary material for: Insights into mycobacteriome composition in Mycobacterium bovis-infected African buffalo (Syncerus caffer) tissue samples
Source: Sci Rep. 2024 Jul 30;14:17537. doi: 10.1038/s41598-024-68189-x (PMC11289279; doi:10.1038/s41598-024-68189-x)
Supplement: Supplementary file 1 — Supplementary Information 1. [file 41598_2024_68189_MOESM1_ESM.pdf]

MinION Mk1C (MC-114017) Final report

30 May 23, 06:56 — 02 Jun 23, 04:20 · 30052023buff3sigdirect · no_sample · MC-114017
Protocol run ID: 0980127d-3e0c-4171-a300-e882e567db99

Run summary | Run configuration | Sequence output | Run health | Run log

Run summary

DATA OUTPUT

Estimated bases	18.81 Gb
Data produced	435.16 GB
Reads generated	23.52 M
Estimated N50	567

RUN DURATION

Elapsed time

69 hours 25 minutes of 72 hours

Run status

Stopped by user

View unit abbreviations used in this report

BASECALLING

Reads called: 100%

Bases called (min Q score: 8): 14.46 Gb Pass | 1.47 Gb Fail

Run configuration

RUN SETUP

Flow cell type	FLO-MIN106
Flow cell type alias	FLO-MIN106
Flow cell ID	FAV36158
Kit type	SQK-NBD112-96

RUN SETTINGS

Specified run length	72 hrs
Active channel selection	On
Pore scan freq.	1.5 hrs
Reserved pores	On
Minimum read length	200 bp
Read splitting	On
Basecalling	Fast model
Modified basecalling	Off
Trim barcodes	Off
Mid-read barcode filtering	Off

DATA OUTPUT SETTINGS

FAST5 output	vbz_compress
FAST5 reads per file	4000
FASTQ output	gzip_compress
FASTQ reads per file	4000
BAM output	Off
Bulk file output	Off
Data location	/data/./30052023buff3sigdirect/no_sample/20230530_0656_MC-114017_FAV36158_0980127d

SOFTWARE VERSIONS

MinKNOW	22.12.5
Bream	7.4.8
Configuration	5.4.7
Guppy	6.4.6
MinKNOW Core	5.4.3

Sequence output

READ LENGTHS · OUTLIERS REMOVED

The read length graph shows the total number of bases vs the read length. The longest 1% of strands are classified as outliers, and excluded to allow focus on the main body of data.

Legend

Basecalled — Estimated

Estimated N50: **567**

% Basecalled: **100%**

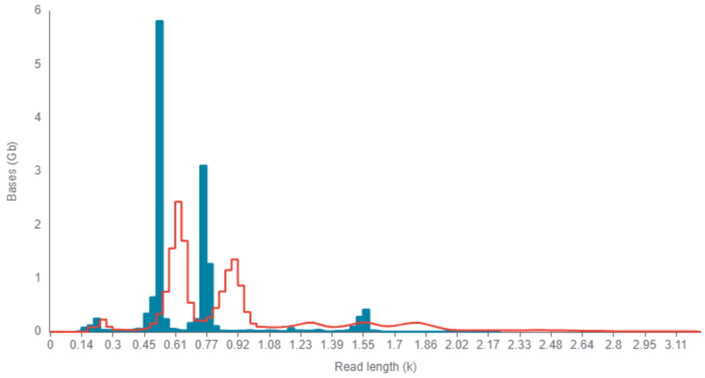

OUTLIERS

The longest 1% of strands are classified as outliers, and aggregated into groups to show their relative amounts.

Read length (kb)	Aggregated reads (Mb)
0 - 64	143.71
64 - 128	0.32
128 - 192	*None*
192 - 256	0.26
256 - 268	0.27

BARCODED READS

The total number of bases for each barcode is calculated and displayed below.

Total bases (Gb)	Barcodes
0–1	barcode01 (Reads: 143167) barcode02 (Reads: 185787) barcode03 (Reads: 223032) barcode04 (Reads: 225580)<br>barcode05 (Reads: 234421) barcode06 (Reads: 345558) barcode07 (Reads: 84789) barcode08 (Reads: 256395) barcode09 (Reads: 291960)<br>barcode10 (Reads: 411909) barcode11 (Reads: 426547) barcode12 (Reads: 219454) barcode13 (Reads: 720581) barcode14 (Reads: 222972)<br>barcode15 (Reads: 204359) barcode16 (Reads: 389719) barcode17 (Reads: 507614) barcode18 (Reads: 438299)<br>barcode19 (Reads: 243830) barcode20 (Reads: 519741) barcode21 (Reads: 600790) barcode22 (Reads: 638707)<br>barcode23 (Reads: 480825) barcode24 (Reads: 401747) barcode25 (Reads: 543477) barcode26 (Reads: 494409)<br>barcode27 (Reads: 637282) barcode28 (Reads: 644290) barcode29 (Reads: 588575) barcode30 (Reads: 570988)<br>barcode31 (Reads: 540071) barcode32 (Reads: 563289) barcode33 (Reads: 711671) barcode34 (Reads: 573163) barcode35 (Reads: 756804)<br>barcode36 (Reads: 1113614) barcode37 (Reads: 676936) barcode38 (Reads: 279384) barcode39 (Reads: 217007)<br>barcode40 (Reads: 263073) barcode41 (Reads: 431716) barcode42 (Reads: 260907) barcode43 (Reads: 257685)<br>barcode44 (Reads: 267674) barcode45 (Reads: 363303) barcode46 (Reads: 246328) barcode47 (Reads: 353936)<br>barcode48 (Reads: 283064) barcode49 (Reads: 376332) barcode50 (Reads: 341726) barcode51 (Reads: 285223)<br>barcode52 (Reads: 353499) barcode53 (Reads: 269599) barcode54 (Reads: 269678) barcode55 (Reads: 293289)<br>barcode56 (Reads: 309239) barcode57 (Reads: 20) barcode58 (Reads: 112) barcode59 (Reads: 8) barcode60 (Reads: 14)<br>barcode61 (Reads: 23) barcode62 (Reads: 12) barcode63 (Reads: 4) barcode64 (Reads: 30) barcode65 (Reads: 8)<br>barcode66 (Reads: 18) barcode67 (Reads: 29) barcode68 (Reads: 5) barcode69 (Reads: 17) barcode70 (Reads: 22)<br>barcode71 (Reads: 13) barcode72 (Reads: 14) barcode73 (Reads: 6) barcode74 (Reads: 11) barcode75 (Reads: 21)<br>barcode76 (Reads: 32) barcode77 (Reads: 5) barcode78 (Reads: 9) barcode79 (Reads: 31) barcode80 (Reads: 10)<br>barcode81 (Reads: 8) barcode82 (Reads: 6) barcode83 (Reads: 49) barcode84 (Reads: 50) barcode85 (Reads: 5)<br>barcode86 (Reads: 10) barcode87 (Reads: 14) barcode88 (Reads: 2) barcode89 (Reads: 19) barcode90 (Reads: 6)<br>barcode91 (Reads: 12) barcode92 (Reads: 2) barcode93 (Reads: 5) barcode94 (Reads: 74) barcode95 (Reads: 9) barcode96 (Reads: 13)

CUMULATIVE OUTPUT

The cumulative output shows the total amount of bases or reads sequenced over time by your device.

Bases

Legend

- **Estimated** — Predicted total number of bases, prior to basecalling
- **Passed** — Bases equal to or above the quality score threshold.
- **Failed** — Bases below the quality score threshold.

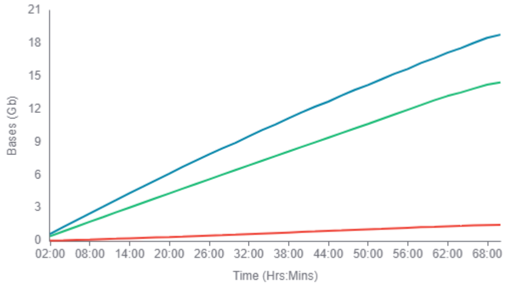

Reads

Legend

- **Total** — Total number of reads, including passed, failed and skipped.
- **Passed** — Reads equal to or above the quality score threshold.
- **Failed** — Reads below the quality score threshold.
- **Skipped** — Reads that will not be basecalled. Post run basecalling is possible.

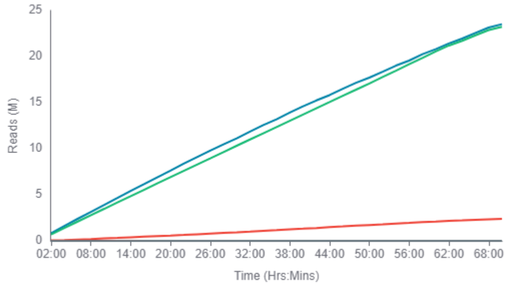

QUALITY SCORE

The quality score is calculated as basecalling is performed on your device. Reads that fall below the minimum value of 8 will be classified as failed reads. You can alter the accepted minimum quality score in MinKNOW.

Legend

— Mode
The most frequent quality score of reads in the run.

Spread
The spread of quality scores, found by calculating full width half maximum.

Min. quality score
Minimum quality score to be accepted as a passed read.

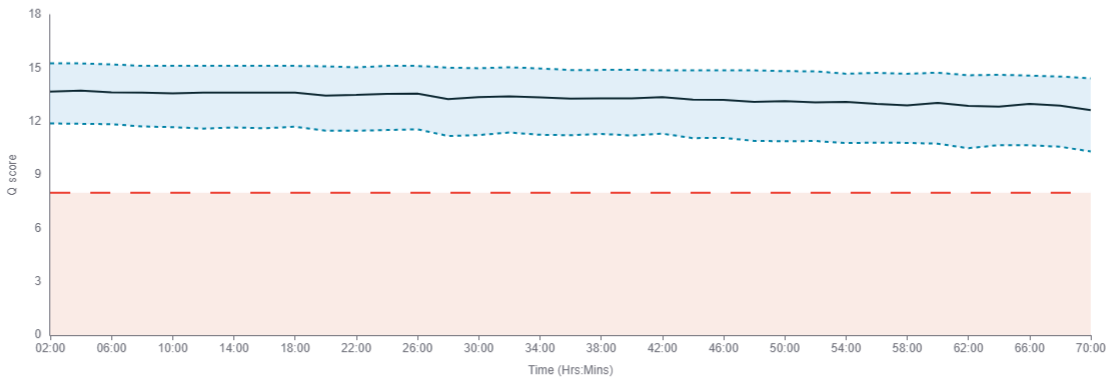

Troubleshooting

Quality score low
This can be due to the translocation speed being out of the accepted range, which can correlate to low quality scores. If you see that the translocation speed is out of the accepted range in the below graph, please see the Flow Cell refuelling page linked here for further troubleshooting.

Run health

PORE ACTIVITY

The Pore activity graph shows the performance of your sample as it is being sequenced during a run.

Legend

- **Sequencing** — Pore currently sequencing
- **Adapter** — Pore currently sequencing adapter
- **Pore available** — Pore available for sequencing
- **Unavailable** — Pore unavailable for sequencing
- **Active feedback** — Channel ejecting analyte
- **No pore** — No pore detected in channel
- **Out of range-high** — Current is positive but unavailable for sequencing
- **Out of range-low** — Current is negative but unavailable for sequencing
- **Multiple** — Multiple pores detected. Unavailable for sequencing.
- **Saturated** — The channel has switched off as current levels exceed hardware limitations
- **Zero** — Pore currently unavailable for sequencing
- **Channel disabled** — Channel is disabled and awaiting another pore scan
- **Unclassified** — Pore status unknown

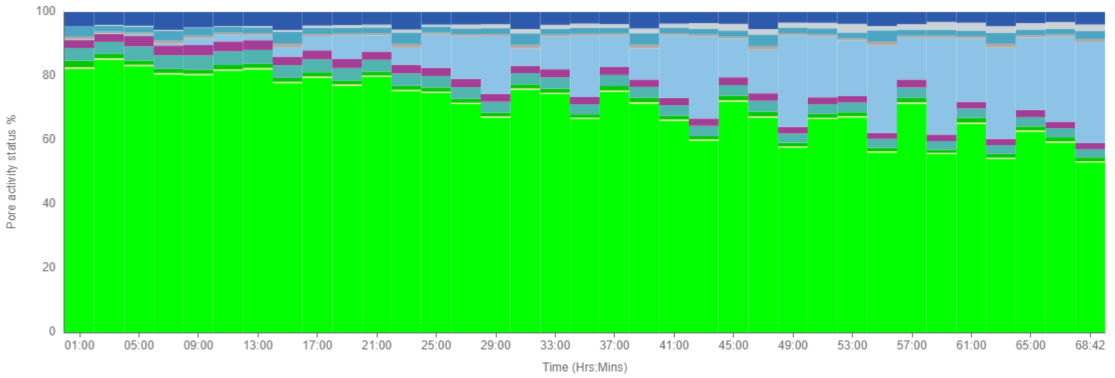

Troubleshooting

General
Some commonly seen issues are excess pores classified as Recovering, Open Pore, or Free Adapter. To find out what advice is applicable for your run, visit the user guide.

PORE SCAN

A Pore scan is performed at configurable time intervals to determine the current status of pores within channels on a Flow Cell. For this run a Pore scan is performed every 1.5 hrs.

Legend

- **Pore available** — Pore in channel available for sequencing
- **Reserved pore** — Pore in reserve, will return to available when required
- **Unavailable** — Pore inhibited from sequencing
- **Saturated** — Possible contamination in the sample
- **Zero** — No current is passing through this pore, possibly due to bubbles on the membrane
- **Inactive** — Pore no longer suitable for further sequencing

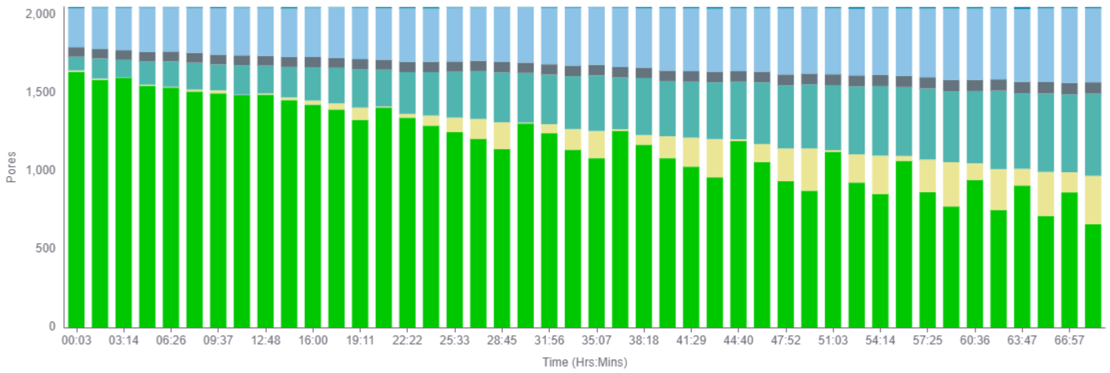

Troubleshooting

High proportion Unavailable
Possible contaminants in library blocking the pore. Consider using the Flow Cell Wash Kit, and reloading a library.

High proportion Inactive
If localised to one area of the Flow Cell, this could indicate that an air bubble has been introduced during the flushing/loading steps. If inactivity is spread across the Flow Cell this could be caused by improper loading of the library, please refer to the user guide for further support.

TRANSLOCATION SPEED

The translocation speed is the rate at which DNA/RNA travels through pores as it is being sequenced.

Legend

— Median --- 75% quartile --- 25% quartile ☐ Accepted range

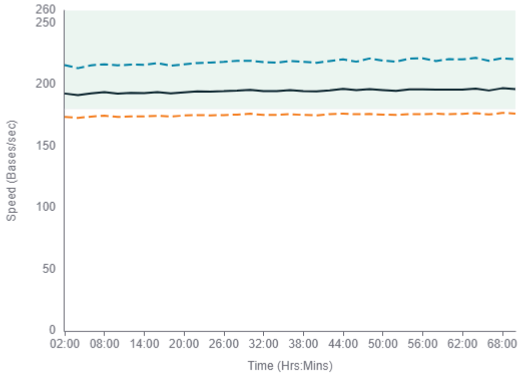

Troubleshooting

Low speed
Check that the Flow Cell is within the target temperature range.

Note
Low-quality and short reads are not included in this graph.

TEMPERATURE

The temperature of the Flow Cell over the run time.

Legend

— Measured — Target

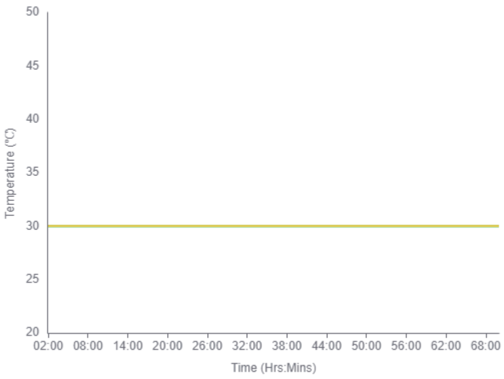

Troubleshooting

Out of range
Check that the Flow Cell is correctly seated and firmly pushed down into the device. Ensure ambient temperature is always within the specified range for your device in the user guide.

Air flow should be good but not excessive. Excessive amounts of cool air blowing on the device could prevent it from reaching target temperature.

Run log

SYSTEM MESSAGES

System messages are a record of the events that occurred in the time covered by this report.

Errors

None

Warnings

Disk usage alert · 01 Jun 23, 10:49
Disk usage alert – you only have 197 GB of space free, which is insufficient for the run. Please free up some space, otherwise your run will stop in approximately 1d 1h 58m.

Events

Disk space · 30 May 23, 06:56
Disk /data has 535 GB space remaining

Waiting for temperature · 30 May 23, 06:56
Waiting up to 300 seconds for temperature to stabilise at 30.0°C

Starting · 30 May 23, 06:56
Starting sequencing procedure

Pore scan starting · 30 May 23, 06:56
Performing Pore Scan

Pore scan result · 30 May 23, 07:00
Pore scan for flow cell FAV36158 has found a total of 1642 pores. 511 pores available for immediate sequencing

Pore scan starting · 30 May 23, 08:31
Performing Pore Scan

Pore scan result · 30 May 23, 08:36
Pore scan for flow cell FAV36158 has found a total of 1590 pores. 512 pores available for immediate sequencing

Pore scan starting · 30 May 23, 10:07
Performing Pore Scan

Pore scan result · 30 May 23, 10:11
Pore scan for flow cell FAV36158 has found a total of 1597 pores. 510 pores available for immediate sequencing

Pore scan starting · 30 May 23, 11:42
Performing Pore Scan

Pore scan result · 30 May 23, 11:47
Pore scan for flow cell FAV36158 has found a total of 1551 pores. 509 pores available for immediate sequencing

Pore scan starting · 30 May 23, 13:18
Performing Pore Scan

Pore scan result · 30 May 23, 13:23
Pore scan for flow cell FAV36158 has found a total of 1538 pores. 509 pores available for immediate sequencing

Pore scan starting · 30 May 23, 14:54
Performing Pore Scan

Pore scan result · 30 May 23, 14:58
Pore scan for flow cell FAV36158 has found a total of 1520 pores. 502 pores available for immediate sequencing

Pore scan starting · 30 May 23, 16:29
Performing Pore Scan

Pore scan result · 30 May 23, 16:34
Pore scan for flow cell FAV36158 has found a total of 1515 pores. 498 pores available for immediate sequencing

Pore scan starting · 30 May 23, 18:05
Performing Pore Scan

Pore scan result · 30 May 23, 18:10
Pore scan for flow cell FAV36158 has found a total of 1487 pores. 505 pores available for immediate sequencing

Pore scan starting · 30 May 23, 19:41
Performing Pore Scan

Pore scan result · 30 May 23, 19:45
Pore scan for flow cell FAV36158 has found a total of 1495 pores. 503 pores available for immediate sequencing

Pore scan starting · 30 May 23, 21:16
Performing Pore Scan

Pore scan result · 30 May 23, 21:21
Pore scan for flow cell FAV36158 has found a total of 1470 pores. 494 pores available for immediate sequencing

Pore scan starting · 30 May 23, 22:52
Performing Pore Scan

Pore scan result · 30 May 23, 22:56
Pore scan for flow cell FAV36158 has found a total of 1450 pores. 490 pores available for immediate sequencing

Pore scan starting · 31 May 23, 00:28
Performing Pore Scan

Pore scan result · 31 May 23, 00:32
Pore scan for flow cell FAV36158 has found a total of 1432 pores. 482 pores available for immediate sequencing

Pore scan starting · 31 May 23, 02:03
Performing Pore Scan

Pore scan result · 31 May 23, 02:08
Pore scan for flow cell FAV36158 has found a total of 1405 pores. 461 pores available for immediate sequencing

Pore scan starting · 31 May 23, 03:39
Performing Pore Scan

Pore scan result · 31 May 23, 03:43
Pore scan for flow cell FAV36158 has found a total of 1412 pores. 499 pores available for immediate sequencing

Pore scan starting · 31 May 23, 05:14
Performing Pore Scan

Pore scan result · 31 May 23, 05:19
Pore scan for flow cell FAV36158 has found a total of 1365 pores. 480 pores available for immediate sequencing

Pore scan starting · 31 May 23, 06:50
Performing Pore Scan

Pore scan result · 31 May 23, 06:55
Pore scan for flow cell FAV36158 has found a total of 1354 pores. 459 pores available for immediate sequencing

Pore scan starting · 31 May 23, 08:26
Performing Pore Scan

Pore scan result · 31 May 23, 08:30
Pore scan for flow cell FAV36158 has found a total of 1341 pores. 449 pores available for immediate sequencing

Pore scan starting · 31 May 23, 10:01
Performing Pore Scan

Pore scan result · 31 May 23, 10:06
Pore scan for flow cell FAV36158 has found a total of 1333 pores. 431 pores available for immediate sequencing

Pore scan starting · 31 May 23, 11:37
Performing Pore Scan

Pore scan result · 31 May 23, 11:41

Pore scan for flow cell FAV36158 has found a total of 1311 pores. 408 pores available for immediate sequencing

Pore scan starting · 31 May 23, 13:13
Performing Pore Scan

Pore scan result · 31 May 23, 13:17
Pore scan for flow cell FAV36158 has found a total of 1309 pores. 496 pores available for immediate sequencing

Pore scan starting · 31 May 23, 14:48
Performing Pore Scan

Pore scan result · 31 May 23, 14:53
Pore scan for flow cell FAV36158 has found a total of 1299 pores. 468 pores available for immediate sequencing

Pore scan starting · 31 May 23, 16:24
Performing Pore Scan

Pore scan result · 31 May 23, 16:28
Pore scan for flow cell FAV36158 has found a total of 1268 pores. 426 pores available for immediate sequencing

Pore scan starting · 31 May 23, 17:59
Performing Pore Scan

Pore scan result · 31 May 23, 18:04
Pore scan for flow cell FAV36158 has found a total of 1256 pores. 391 pores available for immediate sequencing

Pore scan starting · 31 May 23, 19:35
Performing Pore Scan

Pore scan result · 31 May 23, 19:39
Pore scan for flow cell FAV36158 has found a total of 1267 pores. 494 pores available for immediate sequencing

Pore scan starting · 31 May 23, 21:10
Performing Pore Scan

Pore scan result · 31 May 23, 21:15
Pore scan for flow cell FAV36158 has found a total of 1230 pores. 456 pores available for immediate sequencing

Pore scan starting · 31 May 23, 22:46
Performing Pore Scan

Pore scan result · 31 May 23, 22:50
Pore scan for flow cell FAV36158 has found a total of 1222 pores. 413 pores available for immediate sequencing

Pore scan starting · 01 Jun 23, 00:22
Performing Pore Scan

Pore scan result · 01 Jun 23, 00:26
Pore scan for flow cell FAV36158 has found a total of 1213 pores. 394 pores available for immediate sequencing

Pore scan starting · 01 Jun 23, 01:57
Performing Pore Scan

Pore scan result · 01 Jun 23, 02:02
Pore scan for flow cell FAV36158 has found a total of 1204 pores. 356 pores available for immediate sequencing

Pore scan starting · 01 Jun 23, 03:33
Performing Pore Scan

Pore scan result · 01 Jun 23, 03:37
Pore scan for flow cell FAV36158 has found a total of 1203 pores. 490 pores available for immediate sequencing

Pore scan starting · 01 Jun 23, 05:08
Performing Pore Scan

Pore scan result · 01 Jun 23, 05:13
Pore scan for flow cell FAV36158 has found a total of 1172 pores. 436 pores available for immediate sequencing

Pore scan starting · 01 Jun 23, 06:44
Performing Pore Scan

Pore scan result · 01 Jun 23, 06:48
Pore scan for flow cell FAV36158 has found a total of 1144 pores. 367 pores available for immediate sequencing

Pore scan starting · 01 Jun 23, 08:20
Performing Pore Scan

Pore scan result · 01 Jun 23, 08:24
Pore scan for flow cell FAV36158 has found a total of 1144 pores. 342 pores available for immediate sequencing

Pore scan starting · 01 Jun 23, 09:55
Performing Pore Scan

Pore scan result · 01 Jun 23, 10:00
Pore scan for flow cell FAV36158 has found a total of 1133 pores. 482 pores available for immediate sequencing

Pore scan starting · 01 Jun 23, 11:31
Performing Pore Scan

Pore scan result · 01 Jun 23, 11:35
Pore scan for flow cell FAV36158 has found a total of 1106 pores. 393 pores available for immediate sequencing

Pore scan starting · 01 Jun 23, 13:06
Performing Pore Scan

Pore scan result · 01 Jun 23, 13:11
Pore scan for flow cell FAV36158 has found a total of 1098 pores. 345 pores available for immediate sequencing

Pore scan starting · 01 Jun 23, 14:42
Performing Pore Scan

Pore scan result · 01 Jun 23, 14:46
Pore scan for flow cell FAV36158 has found a total of 1096 pores. 471 pores available for immediate sequencing

Pore scan starting · 01 Jun 23, 16:17
Performing Pore Scan

Pore scan result · 01 Jun 23, 16:22
Pore scan for flow cell FAV36158 has found a total of 1073 pores. 377 pores available for immediate sequencing

Pore scan starting · 01 Jun 23, 17:52
Performing Pore Scan

Pore scan result · 01 Jun 23, 17:57
Pore scan for flow cell FAV36158 has found a total of 1056 pores. 327 pores available for immediate sequencing

Pore scan starting · 01 Jun 23, 19:28
Performing Pore Scan

Pore scan result · 01 Jun 23, 19:32
Pore scan for flow cell FAV36158 has found a total of 1049 pores. 437 pores available for immediate sequencing

Pore scan starting · 01 Jun 23, 21:03
Performing Pore Scan

Pore scan result · 01 Jun 23, 21:08
Pore scan for flow cell FAV36158 has found a total of 1012 pores. 338 pores available for immediate sequencing

Pore scan starting · 01 Jun 23, 22:39
Performing Pore Scan

Pore scan result · 01 Jun 23, 22:43
Pore scan for flow cell FAV36158 has found a total of 1014 pores. 427 pores available for immediate sequencing

Pore scan starting · 02 Jun 23, 00:14
Performing Pore Scan

Pore scan result · 02 Jun 23, 00:19
Pore scan for flow cell FAV36158 has found a total of 994 pores. 318 pores available for immediate sequencing

Pore scan starting · 02 Jun 23, 01:50
Performing Pore Scan

Pore scan result · 02 Jun 23, 01:54
Pore scan for flow cell FAV36158 has found a total of 992 pores. 416 pores available for immediate sequencing

Pore scan starting · 02 Jun 23, 03:25
Performing Pore Scan

Pore scan result · 02 Jun 23, 03:30

Pore scan for flow cell FAV36158 has found a total of 969 pores. 299 pores available for immediate sequencing

UNIT ABBREVIATIONS

Byte	B
Kilobyte	KB
Megabyte	MB
Gigabyte	GB
Terabyte	TB

Base	b
Kilobase	kb
Megabase	Mb
Gigabase	Gb
Terabase	Tb

Minutes	mins
Hours	hrs
