# Supplementary material for: Insights into mycobacteriome composition in Mycobacterium bovis-infected African buffalo (Syncerus caffer) tissue samples
Source: Sci Rep. 2024 Jul 30;14:17537. doi: 10.1038/s41598-024-68189-x (PMC11289279; doi:10.1038/s41598-024-68189-x)
Supplement: Supplementary file 2 — Supplementary Information 2. [file 41598_2024_68189_MOESM2_ESM.pdf]

MinION Mk1C (MC-114017) Final report

Oct 31, 23, 10:34 AM UTC+2:00 — Nov 1, 23, 7:58 AM UTC+2:00 · 20231031flonglegyrbuffHiP · no_sample · MC-114017
Protocol run ID: af990f6f-af77-4b14-b66b-90ad1094d168

Run summary | Run configuration | Sequence output | Run health | Run log

Run summary

DATA OUTPUT

Estimated bases	151.53 Mb
Reads generated	536.75 k
Estimated N50	229
Total data produced (pass / fail)	2.48 GB

RUN DURATION

Run time

21 hrs 22 mins / 24 hrs 0 mins (est)

Elapsed time Run limit

Run status

STOPPED · *By user*

View unit abbreviations used in this report

BASECALLING

Reads called	100%
Bases called (min Q score: 9)	116.87 Mb Pass / 20.22 Mb Fail

Run configuration

RUN SETUP

Flow cell type	FLO-FLG114
Flow cell ID	APK571
Kit type	SQK-NBD114-96
Flongle adapter ID	FA-08678

RUN SETTINGS

Run limit	24 hrs
Active channel selection	On
Pore scan freq.	1.5 hrs
Reserved pores	Off
Minimum read length	20 bp
Read splitting	On
Basecalling	High-accuracy model, 400 bps
Modified basecalling	Off
Trim barcodes	Off
Mid-read barcode filtering	Off

DATA OUTPUT SETTINGS

FAST5 output	Off
FASTQ output	gzip_compress
FASTQ reads per file	1000
BAM output	Off
Bulk file output	Off
Data location	/data/./20231031flonglegyrbuffHiP/no_sample/20231031_0834_MC-114017_APK571_af990f6f

SOFTWARE VERSIONS

MinKNOW	23.07.12
Bream	7.7.6
Configuration	5.7.11
Guppy	7.1.4
MinKNOW Core	5.7.5

Sequence output

READ LENGTHS · OUTLIERS REMOVED

The read length graph shows the total number of bases vs the read length. The longest 1% of strands are classified as outliers, and excluded to allow focus on the main body of data.

Legend

Basecalled — Estimated

Estimated N50: **229**

% Basecalled: **100%**

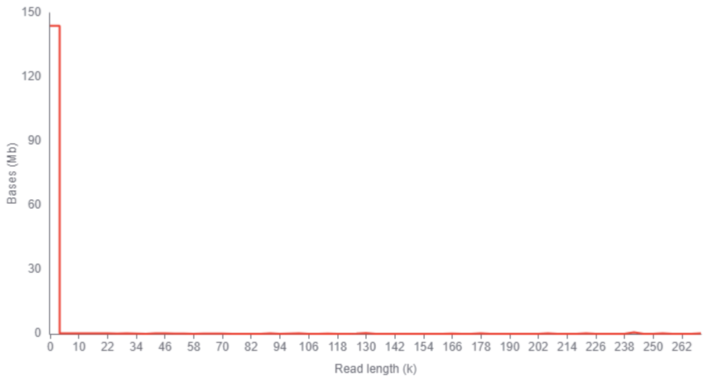

OUTLIERS

The longest 1% of strands are classified as outliers, and aggregated into groups to show their relative amounts.

Read length (kb)	Aggregated reads (Mb)
1 - 3	1.05
3 - 5	0.08
5 - 7	0.02
7 - 9	0.01
9 - 9.25	0.01

BARCODES

Detected barcodes Bases graph Reads graph

Detected barcodes

The total number of bases and reads for each barcode detected are displayed in table below.
Reads/bases must have a quality score above 9 to pass.

Unclassified data

75.98 k (13.3%) — Reads

20.55 Mb (15%) — Bases

Search barcodes

Export CSV

Barcode	Total bases (Mb)	Passed bases (%)	Total reads (k)	Passed reads (%)
barcode01	0.0029	5	0.012	
barcode02	0.0018	24.5	0.008	
barcode03	0.0013	31.7	0.006	3
barcode04	0.0012	15.9	0.006	1
barcode05	0.0013	0	0.006	
barcode06	0.00082	58.6	0.004	
barcode07	0.0012	0	0.006	
barcode08	0.00045	0	0.002	
barcode09	0.00083	22.9	0.004	
barcode10	0.0015	14.4	0.006	1
barcode11	0.00072	63.6	0.003	6

First Prev 1 2 3 4 5 Next Last

CUMULATIVE OUTPUT

The cumulative output shows the total amount of bases or reads sequenced over time by your device.

Bases

Legend

- **Estimated** — Predicted total number of bases, prior to basecalling
- **Passed** — Bases equal to or above the quality score threshold.
- **Failed** — Bases below the quality score threshold.

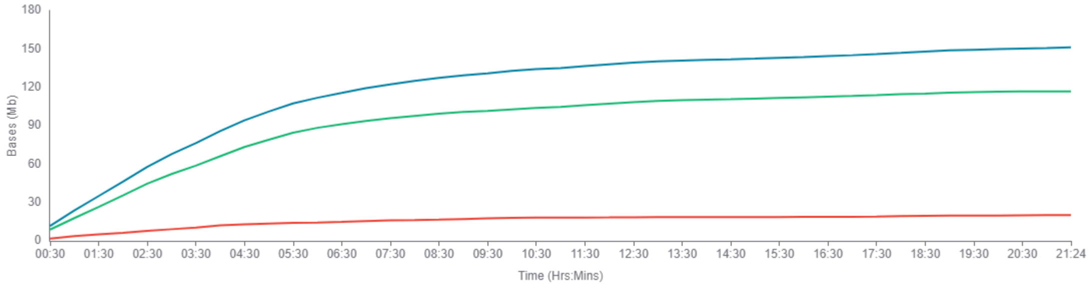

Reads

Legend

- **Total** — Total number of reads, including passed, failed and skipped.
- **Passed** — Reads equal to or above the quality score threshold.
- **Failed** — Reads below the quality score threshold.
- **Skipped** — Reads that will not be basecalled. Post run basecalling is possible.

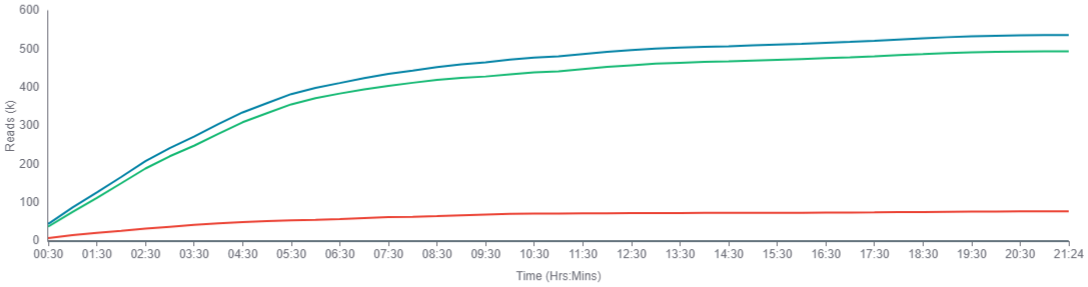

QUALITY SCORE

The quality score is calculated as basecalling is performed on your device. Reads that fall below the minimum value of 9 will be classified as failed reads. You can alter the accepted minimum quality score in MinKNOW.

Legend

- Mode — The most frequent quality score of reads in the run.
- Spread — The spread of quality scores, found by calculating full width half maximum.
- Min. quality score — Minimum quality score to be accepted as a passed read.

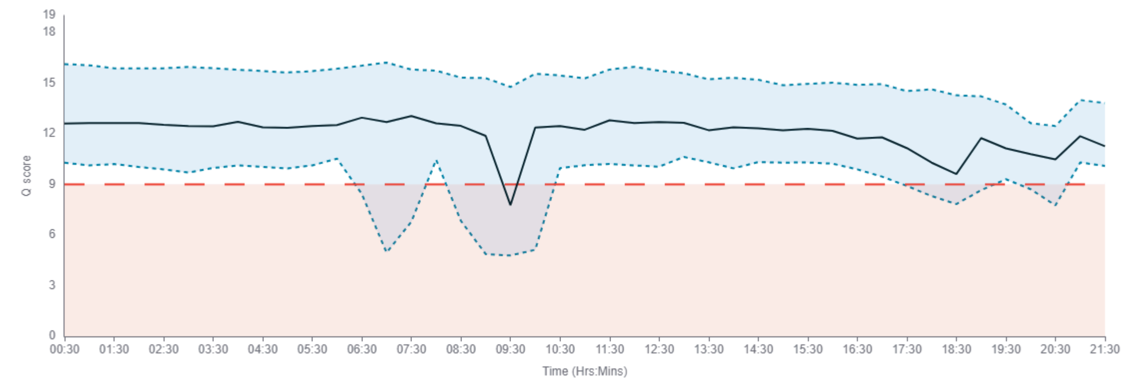

Troubleshooting

Quality score low
This can be due to the translocation speed being out of the accepted range, which can correlate to low quality scores. If you see that the translocation speed is out of the accepted range in the below graph, please see the Flow Cell refuelling page linked here for further troubleshooting.

Run health

PORE ACTIVITY

The Pore activity graph shows the performance of your sample as it is being sequenced during a run.

☑ Show grouped

Legend

- Sequencing — Pore currently sequencing
- Pore available — Pore available for sequencing
- Unavailable — Pore currently unavailable for sequencing
- Inactive — Pore no longer suitable for further sequencing
- Unclassified — Pore status unknown

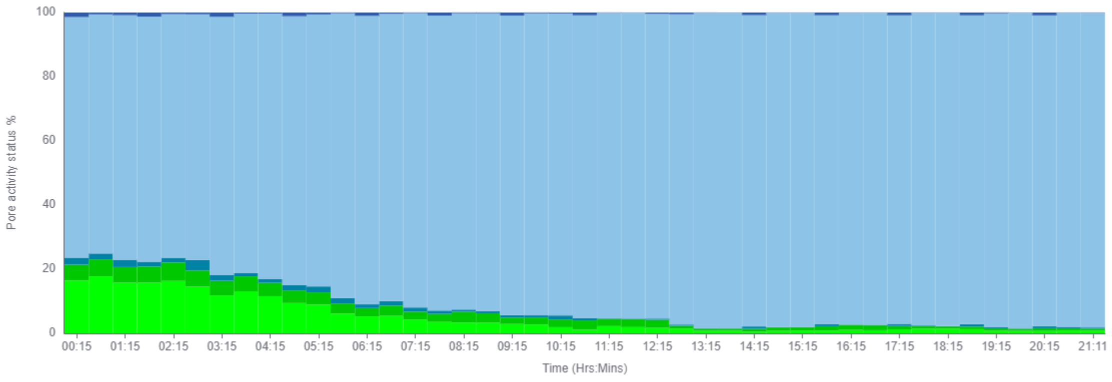

Troubleshooting

General
Some commonly seen issues are excess pores classified as Recovering, Open Pore, or Free Adapter. To find out what advice is applicable for your run, visit the user guide.

PORE SCAN

A Pore scan is performed at configurable time intervals to determine the current status of pores within channels on a Flow Cell. For this run a Pore scan is performed every 1.5 hrs.

Legend

- **Pore available** – Pore in channel available for sequencing
- **Reserved pore** – Pore in reserve, will return to available when required
- **Unavailable** – Pore inhibited from sequencing
- **Saturated** – Possible contamination in the sample
- **Zero** – No current is passing through this pore, possibly due to bubbles on the membrane
- **Inactive** – Pore no longer suitable for further sequencing

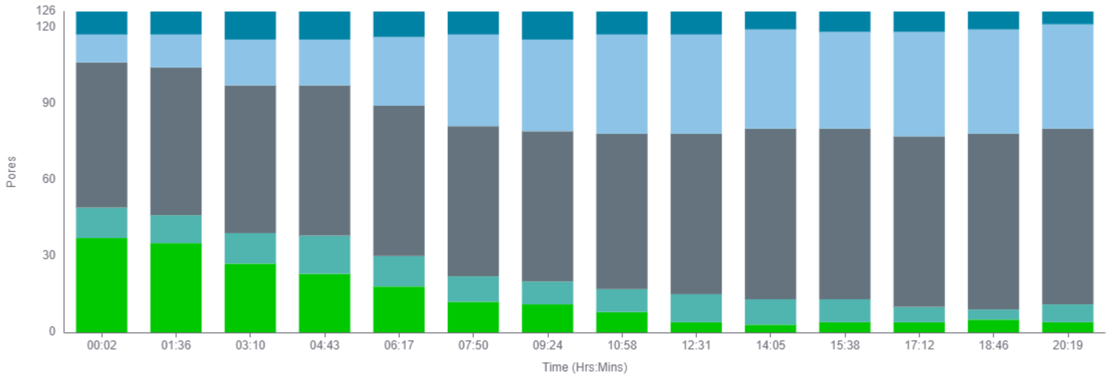

Troubleshooting

High proportion Unavailable
Possible contaminants in library blocking the pore. Consider using the Flow Cell Wash Kit, and reloading a library.

High proportion Inactive
If localised to one area of the Flow Cell, this could indicate that an air bubble has been introduced during the flushing/loading steps. If inactivity is spread across the Flow Cell this could be caused by improper loading of the library, please refer to the user guide for further support.

TRANSLOCATION SPEED

The translocation speed is the rate at which DNA/RNA travels through pores as it is being sequenced.

Legend

— Median --- 75% quartile --- 25% quartile ☐ Accepted range

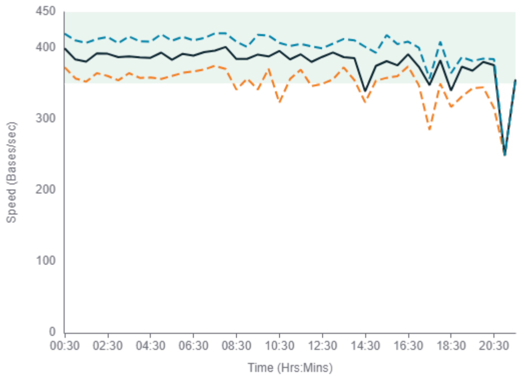

Troubleshooting

Low speed
Check that the Flow Cell is within the target temperature range.

Note
Low-quality and short reads are not included in this graph.

TEMPERATURE

The temperature of the Flow Cell over the run time.

Legend

— Measured — Target

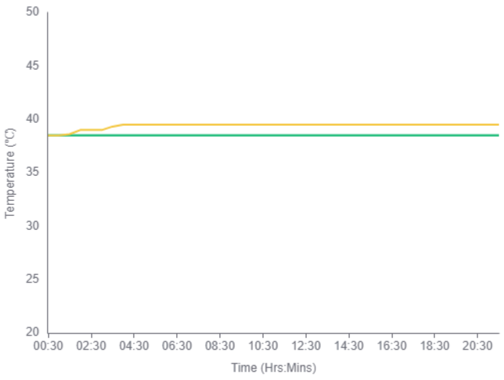

Troubleshooting

Out of range
Check that the Flow Cell is correctly seated and firmly pushed down into the device. Ensure ambient temperature is always within the specified range for your device in the user guide.

Air flow should be good but not excessive. Excessive amounts of cool air blowing on the device could prevent it from reaching target temperature.

Run log

SYSTEM MESSAGES

System messages are a record of the events that occurred in the time covered by this report.

Errors

None

Warnings

None

Events

Disk space · 31 Oct 23, 08:34
Disk /data has 254 GB space remaining

Waiting for temperature · 31 Oct 23, 08:34
Waiting up to 300 seconds for temperature to stabilise at 38.5°C

Starting · 31 Oct 23, 08:35
Starting sequencing procedure

Message · 31 Oct 23, 08:35
Performing Channel Scan

Message · 31 Oct 23, 08:38
Channel scan for flow cell APK571 has found a total of 37 pores. 37 pores available for immediate sequencing

Message · 31 Oct 23, 09:58
Setting temperature to reach 39.1°C

Message · 31 Oct 23, 10:09
Performing Channel Scan

Message · 31 Oct 23, 10:11
Channel scan for flow cell APK571 has found a total of 35 pores. 35 pores available for immediate sequencing

Message · 31 Oct 23, 11:42
Performing Channel Scan

Message · 31 Oct 23, 11:45
Channel scan for flow cell APK571 has found a total of 27 pores. 27 pores available for immediate sequencing

Message · 31 Oct 23, 11:46
Setting temperature to reach 39.5°C

Message · 31 Oct 23, 13:16
Performing Channel Scan

Message · 31 Oct 23, 13:19
Channel scan for flow cell APK571 has found a total of 23 pores. 23 pores available for immediate sequencing

Message · 31 Oct 23, 14:50
Performing Channel Scan

Message · 31 Oct 23, 14:52
Channel scan for flow cell APK571 has found a total of 18 pores. 18 pores available for immediate sequencing

Message · 31 Oct 23, 16:23
Performing Channel Scan

Message · 31 Oct 23, 16:26
Channel scan for flow cell APK571 has found a total of 12 pores. 12 pores available for immediate sequencing

Message · 31 Oct 23, 17:57
Performing Channel Scan

Message · 31 Oct 23, 18:00
Channel scan for flow cell APK571 has found a total of 11 pores. 11 pores available for immediate sequencing

Message · 31 Oct 23, 19:30
Performing Channel Scan

Message · 31 Oct 23, 19:33
Channel scan for flow cell APK571 has found a total of 8 pores. 8 pores available for immediate sequencing

Message · 31 Oct 23, 21:04
Performing Channel Scan

Message · 31 Oct 23, 21:07
Channel scan for flow cell APK571 has found a total of 4 pores. 4 pores available for immediate sequencing

Message · 31 Oct 23, 22:38
Performing Channel Scan

Message · 31 Oct 23, 22:40
Channel scan for flow cell APK571 has found a total of 3 pores. 3 pores available for immediate sequencing

Message · 01 Nov 23, 00:11
Performing Channel Scan

Message · 01 Nov 23, 00:14
Channel scan for flow cell APK571 has found a total of 4 pores. 4 pores available for immediate sequencing

Message · 01 Nov 23, 01:45
Performing Channel Scan

Message · 01 Nov 23, 01:48
Channel scan for flow cell APK571 has found a total of 4 pores. 4 pores available for immediate sequencing

Message · 01 Nov 23, 03:18
Performing Channel Scan

Message · 01 Nov 23, 03:21
Channel scan for flow cell APK571 has found a total of 5 pores. 5 pores available for immediate sequencing

Message · 01 Nov 23, 04:52
Performing Channel Scan

Message · 01 Nov 23, 04:55
Channel scan for flow cell APK571 has found a total of 4 pores. 4 pores available for immediate sequencing

UNIT ABBREVIATIONS

Byte	B	Base	b	Minutes	mins
Kilobyte	KB	Kilobase	kb	Hours	hrs
Megabyte	MB	Megabase	Mb		
Gigabyte	GB	Gigabase	Gb		
Terabyte	TB	Terabase	Tb		

Generated using Run Report Template v.5.7.2
